# Exploring Unsupervised Machine Learning Classification Methods for Physiological Stress Detection

**DOI:** 10.3389/fmedt.2022.782756

**Published:** 2022-03-11

**Authors:** Talha Iqbal, Adnan Elahi, William Wijns, Atif Shahzad

**Affiliations:** ^1^Smart Sensors Lab, Lambe Institute of Translational Research, National University of Ireland Galway, Galway, Ireland; ^2^Electrical and Electronics Engineering, National University of Ireland Galway, Galway, Ireland; ^3^Centre for Systems Modelling and Quantitative Biomedicine, University of Birmingham, Birmingham, United Kingdom

**Keywords:** machine learning, stress monitoring, physiological signals, heart rate, respiratory rate, unsupervised and supervised learning

## Abstract

Over the past decade, there has been a significant development in wearable health technologies for diagnosis and monitoring, including application to stress monitoring. Most of the wearable stress monitoring systems are built on a supervised learning classification algorithm. These systems rely on the collection of sensor and reference data during the development phase. One of the most challenging tasks in physiological or pathological stress monitoring is the labeling of the physiological signals collected during an experiment. Commonly, different types of self-reporting questionnaires are used to label the perceived stress instances. These questionnaires only capture stress levels at a specific point in time. Moreover, self-reporting is subjective and prone to inaccuracies. This paper explores the potential feasibility of unsupervised learning clustering classifiers such as Affinity Propagation, Balanced Iterative Reducing and Clustering using Hierarchies (BIRCH), K-mean, Mini-Batch K-mean, Mean Shift, Density-Based Spatial Clustering of Applications with Noise (DBSCAN) and Ordering Points To Identify the Clustering Structure (OPTICS) for implementation in stress monitoring wearable devices. Traditional supervised machine learning (linear, ensembles, trees, and neighboring models) classifiers require hand-crafted features and labels while on the other hand, the unsupervised classifier does not require any labels of perceived stress levels and performs classification based on clustering algorithms. The classification results of unsupervised machine learning classifiers are found comparable to supervised machine learning classifiers on two publicly available datasets. The analysis and results of this comparative study demonstrate the potential of unsupervised learning for the development of non-invasive, continuous, and robust detection and monitoring of physiological and pathological stress.

## Introduction

There has been a notable increase in depression, anxiety, stress and other stress-related diseases, worldwide (1–3). Stress deteriorates the physical and mental well-being of a human. Particularly, chronic stress leads to a weakened immune system, substance addiction, diabetes, cancer, stroke, and cardiovascular disease (4). Thus, it is of utmost importance to develop robust techniques that can detect and monitor stress continuously, in real-time. The concept of detecting stress is quite complex, as stress has physiological as well as psychological aspects to it. Furthermore, both these aspects are triggered by multiple factors and are difficult to capture (5). The recent development of wearable sensor technology has made it easier to collect different physiological parameters of stress in daily-life.

The use of psychological assessment questionnaires, filled out on different instances in a day, is the most common technique to determine human stress. These questionnaires are limited to capturing stress at a particular time and do not allow continuous as well as real-time stress monitoring (6). The time-bound nature of these questionnaire-based assessments unveils a major problem for the validation of new stress monitoring systems as there is no precise recording of which task or activity caused the participants' stress. To develop an acceptable standard for continuous stress monitoring, Hovsepian et al. (7) used wearable devices and proposed a data-driven stress assessment model, called the *cstress* model. To collect the data in this study, the participants were asked to fill out an Ecological Momentary Assessment (EMA) questionnaire 15 times a day, at random hours. The collected EMA self-report acted as the reference value for stress validation. The *cstress* model compensated for the unpredictable lag that occurred between the stressor and its logging in EMA self-report.

In the literature, several supervised learning algorithms have been utilized for the detection and classification of stress (8–10). These machine learning algorithms include logistic regression, Gaussian Naive Bayes, Decision Tree, Random Forest, AdaBoost, K-Nearest Neighbors, and many others (4). Dalmeida and Masala (11) investigated the role of electrocardiograph (ECG) features derived from heart rate variation (HRV) for the assessment of stress of drivers. A set of different supervised machine learning algorithms were implemented, and the best recall score achieved was 80%. Similarly, Wang and Guo (12) combined the supervised ensemble classifier with an unsupervised learning classifier and used driver's galvanic skin response (GSR) data to detect stress. Their proposed model was able to detect stress with an accuracy of 90.1%.

The physiological parameters that are frequently used for stress analysis are respiratory rate, heart rate, skin conductance, skin temperature, and galvanic skin response (13). As supervised learning requires training labels for training the classifier, in most cases, either the labels are unavailable or inaccurate, in the real-time data collection (14). Several studies have reported the challenges of labeling the stress states and the importance of addressing these issues for the further development of sensor-based stress monitoring systems (15–17). The challenges of poor-quality reference data and human bias encourage the exploration of unsupervised machine learning algorithms for stress detection and monitoring, as the unsupervised algorithms do not require reference data.

## Related Work; Unsupervised Learning Classification

Throughout the literature, most authors are dedicated to the use of techniques based on supervised learning classification while the use of unsupervised learning methods is relatively new in the stress monitoring field. Rescio et al. (18) implemented the k-means clustering algorithm for stress classification using heart rate (HR), galvanic skin response (EDA) and electrooculogram (EOG) signals of 11 volunteers. To induce stress, the participants were asked to perform a mental arithmetic task and complex LEGO assembly without instruction. Authors have reported the classification accuracy of 70.6% with heart rate, 74.6% with EDA and 63.7% with EOG used as a single variable unsupervised classification model. Huysmans et al. (5) proposed a Self-Organizing Maps (SOM) based mental stress detection model that uses skin conductance (SC) and the electrocardiogram (ECG) of the test subjects. The authors recruited a group of 12 subjects and asked them to complete three stress-related tasks (each of 2 min). The first task was the Stroop Word Color test, in which subjects had to select the color of the word rather than the written word. The second task was the mental arithmetic task, in which the subjects had to count backwards from 1,081 with the difference of 7. The final task was to talk about a common stressful event that ever happened to them. The authors reported the average test accuracy of 79.0% using the proposed SOM based classifier. Ramos et al. (19) used Naïve Bayes and logistic regression models to classify the stress outside the laboratory settings. They collected the heart rate, breathing rate, skin temperature and acceleration data from 20 volunteers while they were performing physical activity (such as walking, cycling, or sitting). To induce stress, the authors used random noises, verbal mathematical questions, and a cold-water test. The activity data was ignored and an accuracy of 65% was achieved by the authors. Maaoui et al. (17) investigated the use of three unsupervised learning classification methods [K-mean, Gaussian Mixture Model (GMM), and SOM] to determine the stress levels using a low-cost webcam. Along with the webcam, the authors also collected the heart rate (extracted seven attributes) of 12 students volunteers. The authors reported the classification error rate of the three algorithms as 13.05% (K-means), 44.04% (GMM) and 36.57% (SOM) classifier. Similarly, Fiorini et al. (20) compared the performance of three unsupervised classification techniques (K-means, K-medoids, and SOM) with three supervised learning techniques [Support Vector Machine (SVM), Decision Tree (DT), and K-nearest neighbors (K-NN)]. They collected ECG, EDA, and electric brain activity signals of 15 healthy individuals. The authors designed the study to induce three different emotional states (i.e., relax, positive, and negative) by the means of social interaction. The reported classification accuracy for the best-performing unsupervised classifier (K-means) was 77% while for the same model the best-performing supervised classifier (K-NN) was 85%.

This paper explores the possible use of unsupervised classification methods for physiological stress detection. To perform a comparative analysis of the performance of unsupervised learning algorithms against supervised learning algorithms, two publicly available datasets were used. A total of seven most common supervised and seven unsupervised learning algorithms were implemented in Python Programming Language. The implemented unsupervised algorithms are Affinity Propagation (21), Balanced Iterative Reducing and Clustering using Hierarchies (BIRCH) (22), K-Mean, Mini-Batch K-Mean (23), Mean Shift, Density-Based Spatial Clustering of Applications with Noise (DBSCAN) (24) and Ordering Points To Identify the Clustering Structure (OPTICS) (25). For comparison, supervised learning algorithms such as logistic regression, Gaussian naïve Bayes, decision tree, random forest, AdaBoost and K-nearest neighbors, are implemented.

## Materials and Methods

To address the challenge of manual annotation and labeling of the physiological signal as stress or non-stress in a supervised learning setup, we investigated the efficiency of the commonly used unsupervised machine learning algorithms, illustrated in the literature. For assessment of the efficiency of these methods and comparative analysis, two publicly available datasets were downloaded. The first dataset is provided by the Massachusetts Institute of Technology (MIT), named Stress Recognition in Automobile Drivers by Healey (26), and is available on Physionet, while the second dataset is called the SWELL-KW dataset, available on Kaggle (27). Both the datasets contain heart rate variation features and provide labeled heart rate and respiratory rate parameters. The efficiencies of the supervised and unsupervised learning algorithms were benchmarked and are provided using standard measures of accuracy, precision, recall, and F1-score matrices of each classifier.

### Performance Assessment Matrices

The performance of the classifier is assessed using the following metrics:

- The accuracy of a classifier is defined as the percentage of total correctly predicted labels in the test dataset, given mathematically as (equation 1):
  (1) $$\begin{array}{l} \text{Accuracy }=\;\frac{\text{true positive labels }+\text{ true negative labels}}{\text{total readings}} \end{array}$$
- The precision and recall are calculated using equations 2 and 3:
  (2) $$\begin{array}{l} \text{Precision }=\;\frac{\text{true positive labels}}{\text{true positive labels }+\text{ false positive labels}} \end{array}$$
  (3) $$\begin{array}{l} \text{Recall }=\;\frac{\text{true positive labels}}{\text{true positive labels }+\text{ false negative labels}} \end{array}$$
- The F1-score of a classifier is the harmonic mean of its precision and recall. Equation 4 shows how F1-score is calculated, mathematically:
  (4) $$\begin{array}{l} \text{F}1-\text{Score }=\;2\;*\frac{\text{Precision }*\text{Recall}}{\text{Precision }+\text{ Recall}} \end{array}$$

### Data Collection

To explore the usability of unsupervised machine learning classifiers in stress monitoring and comparison with supervised learning methods, two publicly available datasets were downloaded. Details of both datasets are described below.

#### Stress Recognition in Automobile Drivers Dataset

The dataset is developed by Healey (28, 29) during her PhD program at MIT. The dataset consists of the electrocardiogram (ECG), galvanic skin response (GSR), electromyogram (EMG), respiratory rate, and heart rate measured using wearable sensors along with stress/non-stress labels generated from a combination of questionnaires and captured videos of the drivers. A total of 18 young drivers were asked to drive in different stress-inducing scenarios, such as at highways, rush hours and red lights, as well as a non-stress scenario (marked as non-stress or baseline readings). To rate the driver's stress levels, three different methods were used. These methods included self-reporting questionnaires, experimental design and metrics defined by independent annotators based on the video recording of the drivers. The dataset has baseline reading along with three different stress level readings (low, medium, and high stress).

#### SWELL-KW Dataset

The SWELL-Knowledge Work (SWELL-KW) dataset (27) provides heart rate variability (HRV) indices from sensor data for stress monitoring in an office work environment. The experiment was conducted on 25 subjects, performing typical office work such as preparing presentations, reading emails, and preparing work reports. Three different working conditions were defined by the authors:

- Neutral/no-stress: the subjects were allowed to complete the given task with no time boundary.
- Time pressure (a stress condition): the time to complete the given task was reduced to 2/3 of the time the subject took in the neutral condition.
- Interruption (a stress condition): during this time, subjects received 8 different emails. Some of the emails were related to their task and were asked to take specific action while some emails were not related to their task.

The experiment recorded data of facial expression, computer logging, skin conductance and ECG signal. For labeling, Rating Scale Mental Effort (RSME) (30) and Self-Assessment-Manikin Scale (SAMS) (31) were used. Moreover, all subjects were also asked to report their perceived stress on a 10-point scale (from not-stressed to very stressed) using a visual analog scale.

### Unsupervised Classification Algorithms

Most of the unsupervised classification algorithms are based on clustering algorithms. Clustering algorithms find best suited natural groups within the given feature space. In this study, the sensor data for stress and non-stress states of the participants are considered as the feature vector. The most widely used unsupervised classifiers implemented in this study are introduced in the following subsections.

#### Affinity Propagation

Affinity propagation takes the input data points as a measure of similarity between two data points. Each data point within the dataset sends a message to all other data points about the target relative attractiveness. Once the sender is associated with its target (stress/no-stress), the target becomes an exemplar. All the points with similar exemplars are combined to form one cluster. The classifier finds a set of different exemplars (representative points of each cluster) that best summarizes the data points within the dataset (21).

#### BIRCH Classifier

Balanced Iterative Reducing and Clustering using Hierarchies (BIRCH) classifier constructs tree structure from which classification cluster centroids are obtained. The BIRCH classification algorithm utilizes the tree structure to cluster the input data. The tree structure is called a clustering feature tree (CF Tree). Each node of the tree is made of a clustering feature (CF). The BIRCH clusters multi-dimensional input data entities to produce the best number of clusters with the available memory and time constraints. The algorithm typically finds good clusters within a single scan but can improve the quality with some additional scans (22).

#### K-Mean Classifier

The K-mean classifier is one of the most frequently used, unsupervised learning classifiers. The algorithm assigns the group label to each data point to minimize the overall variance of each cluster (23). The algorithm starts with a random group of centroids, considering each centroid as a cluster, and performs repetitive calculations to adjust the position of centroids. The algorithm stops the optimization of clusters when the centroids are stable (no change in their values) or a defined number of iterations is achieved.

#### Mini-Batch K-Mean Classifier

Mini-Batch K-mean classifier is a modified version of the K-mean classifier. The classifier clusters the dataset using mini-batches of the data points rather than using whole data. This classifier is also robust to statistical noise and performs the classification of a large dataset more quickly (23).

#### Mean Shift Classifier

The mean shift classifier finds the underlying density function and classifies the data based on the density distribution of the data points in feature space (32). The mean shift classification algorithm tries to discover different blobs within a smooth density of the given dataset. The algorithm updates the candidates for centroids that are then considered as the mean of the points with the given region. These candidates are filtered to eliminate near-duplicate centroids to form the final set of centroids, that form the clusters.

#### DBSCAN Classifier

Density-Based Spatial Clustering of Applications with Noise (DBSCAN) finds the highest density areas in the given feature domain and expands those areas, forming clusters of feature space (stress/non-stress) (24). The DBSCAN finds neighborhoods of a data point exceeding a specified density threshold. This threshold is defined by the minimum number of data points required within a radius of the neighborhood (minPts) and the radius of the neighborhood (eps). Both the parameters are initialized manually at the start of the algorithm.

#### OPTICS Classifier

Ordering Points To Identify the Clustering Structure (OPTICS) is derived from the DBSCAN classifier, where a minimum of samples are required as a hyper-parameter to classify the data as a cluster (feature) (25).

### Supervised Classification Algorithms

This study also implemented supervised classifiers, logistic regression, Gaussian naïve Bayes, decision tree, random forest, AdaBoost and K-nearest neighbors for comparison of results with the unsupervised classifiers. All these algorithms are briefly defined below. Interested readers are referred to Chaitra and Kumar (33) for details.

#### Logistic Regression Classifier

Logistic Regression is one of the simplest machine learning algorithms mostly used for binary classification problems. Logistic regression estimates and classifies based on the relationship between independent and dependent binary features within a dataset.

#### Gaussian Naïve Bayes Classifier

The Naive Bayesian classifier is a probabilistic classifier. Naive Bayesian (NB) has only one parent node in its Directed acyclic graphs (DAGs), which is an unobserved node, and have many children nodes, representing observed nodes. NB works with a strong assumption that all the child nodes are independent of their parent node and thus, one may say that Naïve Bayesian classifier is a type of estimator.

#### Decision Tree Classifier

The Decision tree classifies by sorting input instances based on feature values. Each node of the decision tree shows a classified feature from an input instance while each branch shows an assumed nodal value. Classification of instances starting from the root and is sorted depending upon their feature values.

#### Random Forest Classifier

The Random Forest is a supervised machine learning algorithm. This algorithm creates random trees (forest) that are somewhat like decision trees and the training method selected is always begging, as in begging learning models are linearly combined to increase the overall accuracy. While growing new trees, random forest adds more randomness to the existing model. Instead of finding the most important target feature for node splitting, this algorithm searches for the best feature in the random subset of target features. In this way, we get wide diversity which in-return results in a better model. So, as random forest only considers a random subset of features for splitting a node, we can make the trees of the model more random by using random thresholding of every feature rather than looking for the best threshold value.

#### AdaBoost Classifier

The boosting refers to a group of techniques that creates a strong classifier using many weak classifiers. To find a weak classifier, different machine learning-based algorithm having varied distribution is used. Each learning algorithm generates a new weak classification rule. This process is iterated many times and at the end, a boosting algorithm is formed by combining all newly generated weak classifiers rules to make a strong rule for prediction. A few steps should be followed for the selection of the right distribution:

- Step 1: Give all the distributions to the base learner and assign equal weights to every observation.
- Step 2: If the first base learner gives any prediction error, then pay more attention to observations causing this prediction error. Then, apply a new base learner.
- Step 3: Until the base learning limit is reached, or the desired accuracy is achieved, keep repeating Step 2.

#### K-Nearest Neighbors Classifier

The k-Nearest Neighbor (kNN) is one of the simplest instance-based learning algorithms. Working of kNN is as follows. It classifies all the proximity instances, in a database, into a single group and then when a new instance (feature) comes, the classifier observes the properties of the instance and places it into the closest matched group (nearest neighbor). For accurate classification, initializing a value to k is the most critical step in the kNN classifier.

## Results and Discussions

**Figure 1:**
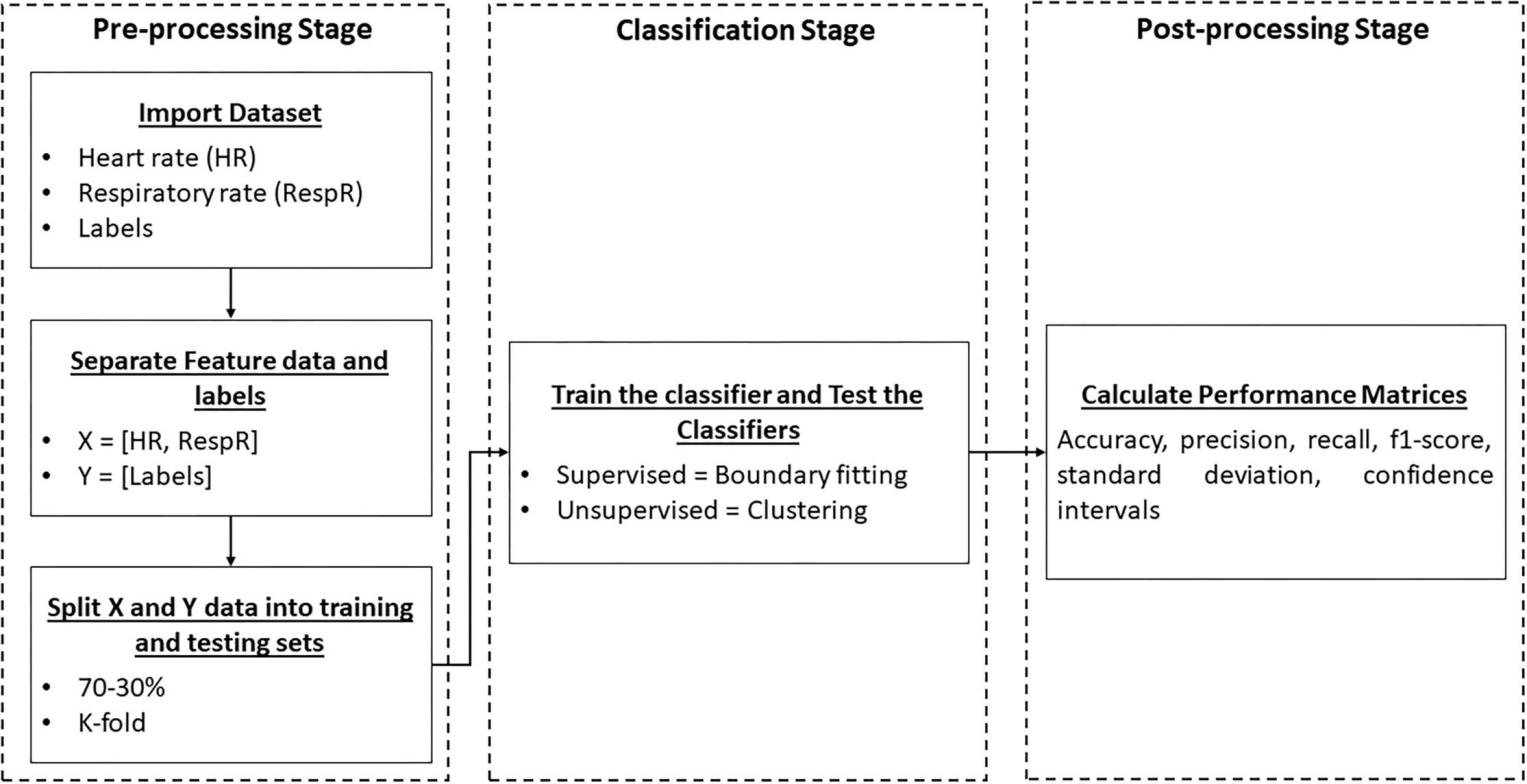
Block diagram of the implemented classification methods illustrating pre-processing, classification, and post-processing stages.

All the algorithms are implemented in python using the scikit learn library. Table 1 shows the hyper-parameter settings of all the classifiers discussed above. Figure 1 demonstrates the overall steps involved in the implementation of the supervised and unsupervised classifiers. In the pre-processing stage, the heart rate, respiratory rate, and stress/non-stress label data are accumulated from the dataset. In the second step, the collected data is split into 70-30% or k-folds to have the training and testing sets. In the classification stage, the supervised learning classifiers are trained and tested to classify the input data into stress/non-stress using boundary fitting while in the case of unsupervised learning classifiers, clustering is performed on the input data and two clusters are formed corresponding to stress and non-stress data. In the final stage (post-processing), different performance evaluation metrics (accuracy, recall, precision, f1-score, standard deviation, and 95% confidence intervals) are calculated and reported.

**Table 1:** Hyper-parameters settings and python library used for implementation.

**Algorithm type**	**Classifiers**	**Train-test split**	**Hyper-parameters**	**Python library**
Supervised machine learning algorithm	Logistics regression		• Solver = “lbfgs” • Penalty = “l2”	sklearn.linear_model
	Gaussian Naïve Bayes		• Variance smooting = 1e-09	sklearn.naive_bayes
	Decision tree		• Quality of split criterion = “gini” • Value of max_depth was varied between range (1-11 with increment of 1) • Maximum number of features to consider = “auto”	sklearn.tree
	Random forest		• Quality of split criterion = “gini” • Maximum depth of trees = 11 • Maximum number of features to consider = “auto” • Number of trees in the forest = 10	sklearn.ensemble
	AdaBoost		• Learning rate was varied between range (0.01-1.1 with increment of 0.01) • Maximum number of estimators at which boosting is terminated was varied between range (50-200 with increment of 10) • Algorithm = “SAMME.R”	sklearn.ensemble
	K-nearest neighbors		• Number of neighbors required was set to 2	sklearn.neighbors
	K-nearest neighbors	70-30% and 10-fold cross validation	• Number of neighbors required set at 5	sklearn.neighbors
Unsupervised machine learning algorithm	Affinity propagation		• Damping factor was set at 0.8 to maintain current value relative to incoming value (weight 1-damping) • Maximum iteration = 200 • Maximum number of iterations with no change in number of estimated clusters = 15	sklearn.cluster
	BIRCH		• Threshold from which the radius of subcluster should be lesser = 0.5 • Number of clusters = length of unique ids in training set (default = 2)	sklearn.cluster
	DBSCAN		• Maximum distance between two samples for consideration as neighbors (eps) = 0.50 • Minimum samples in neighborhood of a point to consider it as core point = 9 • Distance calculation method = “eulidean”	sklearn.cluster
	K-mean		• Number of neighbors required was set to 2	sklearn.cluster
	Mini-batch K-mean		• Number of neighbors required was set to 2	sklearn.cluster
	Mean shift		• Number of clusters = length of unique ids in training set (default = 2)	sklearn.cluster
	OPTICS		• Maximum distance between two samples for consideration as neighbors (eps) = 0.80 • Minimum samples in neighborhood of a point to consider it as core point = 10	sklearn.cluster

The performance of unsupervised and supervised learning algorithms was tested on the two datasets. The Stress Recognition in Automobile Drivers Dataset was a smaller dataset with 4,129 data points for each feature, i.e., heart rate and respiratory rate, along with stress/non-stress labels. The SWELL-KW dataset was a relatively larger dataset with a total of 204,885 data points for the heart rate feature along with stress/non-stress conditions. Each data point is considered as a separate sample and is selected randomly for test and train sets, for supervised learning classifiers.

In real-time, the unsupervised classifier is fed with control data and asked to classify the data into stress and non-stress condition. Then new data point is passed to the classifier and based on the centroids calculated using the control data, the new data point is placed in a specific cluster. For the comparison, a set of different supervised learning classifiers were also implemented, and the performance of the classifiers was evaluated using classification accuracies, precision, recall, and F1-scoring matrices. The results of the classifiers are discussed below.

### Stress Recognition in Automobile Drivers Dataset

It is a well-known fact that all the traditional machine learning classifiers are data-hungry. As the Stress Recognition in Automobile Drivers dataset is a smaller dataset, the highest classification accuracy achieved (with 70-30% train-test split) using combined heart rate and respiratory rate along with supervised learning algorithm is 66.8% (AdaBoost classifier) while for single feature model, i.e., heart rate and respiratory rate separately, the highest classification accuracy is 61.9% (Decision Tree classifier) and 66.8% (AdaBoost classifier), respectively. These results are better than previously reported accuracy values (52.6 and 62.2% for heart rate and respiratory rate models) (26). Similarly, when combined heart rate and respiratory rate is used along with unsupervised learning classification, the highest classification accuracy achieved is 63.8% (Affinity Propagation classifier). If a single feature model is used, the highest accuracy for the heart rate feature model becomes 59.7% while for the respiratory rate feature model, it is 65% using the Affinity Propagation classifier. K-fold cross-validation (with k = 10) was also performed using supervised learning models. The highest achieved accuracies for a single feature model are 59.9% for heart rate and 63.9% for respiratory rate while two feature models (heart rate and respiratory rate combined) gave an accuracy of 65.6%. Detailed analyses of different supervised and unsupervised learning algorithms are illustrated in Tables 2A,B, 3.

**Table 2A:** Results of supervised learning algorithms on stress recognition in automobile drivers dataset.

**Datasets**	**Classifiers**	**Feature**	**Test-train split**	**Classification accuracy**	**Precision**	**Recall**	**F1-score**
Stress recognition in automobile drivers dataset	Logistic regression	Heart rate and respiratory rate		59.3%	0.59	0.59	0.59
	Gaussian Naive Bayes			56.5%	0.60	0.59	0.59
	Decision tree			63.4%	0.64	0.64	0.63
	Random forest			65.0%	0.65	0.66	0.65
	AdaBoost			66.8%	0.67	0.66	0.65
	KNN = 5			63.7%	0.63	0.63	0.63
	KNN = 2			58.1%	0.60	0.57	0.56
Stress recognition in automobile drivers dataset	Logistic regression	Heart rate		58.4%	0.59	0.58	0.58
	Gaussian Naive Bayes			56.0%	0.59	0.56	0.55
	Decision tree			61.9%	0.66	0.062	0.57
	Random forest		70-30 %	56.2%	0.56	0.56	0.56
	AdaBoost			61.5%	0.61	0.61	0.60
	KNN = 5			54.4%	0.54	0.54	0.54
	KNN = 2			51.7%	0.55	0.52	0.50
Stress recognition in automobile drivers dataset	Logistic regression	Respiratory rate		63.2%	0.70	0.63	0.55
	Gaussian Naive Bayes			63.4%	0.72	0.63	0.55
	Decision tree			62.4%	0.64	0.62	0.63
	Random forest			56.9%	0.57	0.57	0.57
	AdaBoost			66.8%	0.66	0.67	0.67
	KNN = 5			59.5%	0.59	0.60	0.59
	KNN = 2			54.0%	0.58	0.54	0.53

**Table 2B:** Results of supervised learning algorithms on Stress recognition in automobile drivers dataset (K-fold cross validation).

**Datasets**	**Classifiers**	**Feature**	**Test-train split**	**Classification accuracy**	**Standard deviation**	**Confidence limits**	
						**Lower**	**Upper**
Stress recognition in automobile drivers dataset	Logistic regression	Heart rate and respiratory rate		61.5%	0.038	58.8%	64.2%
	Gaussian Naive Bayes			61.6%	0.022	58.9%	64.3%
	Decision tree			64.1%	0.047	61.5%	66.8%
	Random forest			64.0%	0.029	61.3%	66.6%
	AdaBoost			65.6%	0.036	62.9%	68.2%
	KNN = 2			54.9%	0.051	52.2%	57.6%
	KNN = 5			58.6%	0.034	55.9%	61.3%
Stress recognition in automobile drivers dataset	Logistic regression	Heart rate		58.7%	0.20	57.2%	60.2%
	Gaussian Naive Bayes			56.4%	0.024	54.9%	57.9%
	Decision tree			59.9%	0.019	58.4%	61.4%
	Random forest		10-fold cross validation	57.5%	0.027	56.0%	59.0%
	AdaBoost			59.9%	0.016	58.4%	61.4%
	KNN = 5			52.0%	0.023	50.4%	53.5%
	KNN = 5			56.1%	0.024	54.6%	57.6%
Stress recognition in automobile drivers dataset	Logistic regression	Respiratory rate		58.3%	0.037	55.6%	61.0%
	Gaussian Naive Bayes			58.7%	0.038	56.0%	61.4%
	Decision tree			61.4%	0.053	58.7%	64.0%
	Random forest			59.4%	0.50	56.7%	62.1%
	AdaBoost			63.9%	0.036	61.2%	66.5%
	KNN = 2			54.6%	0.039	51.9%	57.4%
	KNN = 5			59.0%	0.052	56.3%	61.7%

**Table 3:** Results of unsupervised learning algorithms on stress recognition in automobile drivers dataset.

**Datasets**	**Classifiers**	**Feature**	**Test-train split**	**Classification accuracy**	**Precision**	**Recall**	**F1-score**
Stress recognition in automobile drivers dataset	Affinity propagation	Heart rate and respiratory rate		63.8%	0.65	0.64	0.62
	BIRCH			54.9%	0.62	0.57	0.50
	DBSCAN			53.8%	0.56	0.54	0.41
	K-mean			55.7%	0.62	0.56	0.52
	Mini-batch K-mean			53.0%	0.28	0.53	0.37
	Mean shift			53.0%	0.28	0.53	0.37
	OPTICS			54.1%	0.54	0.54	0.53
Stress recognition in automobile drivers dataset	Affinity propagation	Heart rate		59.7%	0.60	0.82	0.69
	BIRCH			49.1%	0.66	0.49	0.38
	DBSCAN			54.7%	0.30	0.55	0.39
	K-mean		70-30 %	55.5%	0.61	0.55	0.53
	Mini-batch K-mean			54.8%	0.61	0.55	0.52
	Mean shift			54.7%	0.30	0.55	0.39
	OPTICS			51.6%	0.51	0.52	0.51
Stress recognition in automobile drivers dataset	Affinity propagation	Respiratory rate		65.0%	0.77	0.65	0.57
	BIRCH			57.4%	0.33	0.57	0.42
	DBSCAN			60.6%	0.62	0.61	0.53
	K-mean			59.8%	0.63	0.60	0.60
	Mini-batch K-mean			60.3%	0.6	0.60	0.60
	Mean shift			57.4%	0.33	0.57	0.42
	OPTICS			54.6%	0.49	0.55	0.46

### SWELL-KW Dataset

The results of different supervised and unsupervised learning algorithms using the SWELL-KW dataset are illustrated in Tables 4A,B, 5. The highest classification accuracy achieved (with 70-30% train-test split) using a supervised learning algorithm is 74.8% (Decision Tree/Random Forest classifier), which is better than previously reported results for one physiological modality (accuracy = 64.1%) in (34) while for unsupervised learning is 68.3% (Mean shift classifier). The overall classification accuracies of the supervised classifiers do not change significantly with k-fold cross-validation applied to the data. The highest classification accuracy achieved using 10-fold validation is 75.0%.

**Table 4A:** Results of supervised learning algorithms on SWELL-KW dataset.

**Datasets**	**Classifiers**	**Feature**	**Test-train split**	**Classification accuracy**	**Precision**	**Recall**	**F1-score**
SWELL-KW dataset	Logistic regression	Heart rate	70-30 %	70.2%	0.70	0.70	0.64
	Gaussian naive bayes			70.3%	0.70	0.70	0.64
	Decision tree			74.8%	0.74	0.75	0.73
	Random forest			74.8%	0.74	0.75	0.73
	AdaBoost			74.6%	0.75	0.75	0.71
	KNN = 5			71.8%	0.71	0.72	0.71
	KNN = 2			62.7%	0.68	0.63	0.64

**Table 4B:** Results of supervised learning algorithms on SWELL-KW dataset (K-fold cross validation).

**Datasets**	**Classifiers**	**Feature**	**Test-train split**	**Classification accuracy**	**Standard deviation**	**Confidence limits**	
						**Lower**	**Upper**
SWELL-KW dataset	Logistic regression	Heart rate	10-fold cross validation	70.2%	0.002	70.0%	70.4%
	Gaussian Naive Bayes			70.3%	0.002	70.4%	70.5%
	Decision tree			74.8%	0.002	74.6%	75.0%
	Random forest			75.0%	0.003	74.8%	75.2%
	AdaBoost			74.6%	0.003	74.4%	74.8%
	KNN = 2			62.8%	0.002	62.6%	63.0%
	KNN = 5			72.0%	0.003	71.8%	72.2%

**Table 5:** Results of unsupervised learning algorithms on SWELL-KW dataset.

**Datasets**	**Classifiers**	**Feature**	**Test-train split**	**Classification accuracy**	**Precision**	**Recall**	**F1-score**
SWELL-KW dataset	Affinity propagation	Heart rate	70-30 %	66.5%	0.44	0.67	0.53
	BIRCH			68.1%	0.66	0.68	0.60
	K-mean			66.7%	0.45	0.67	0.53
	Mini-batch K-mean			66.7%	0.45	0.67	0.53
	Mean shift			68.3%	0.69	0.68	0.60
	DBSCAN			66.7%	0.45	0.67	0.53
	OPTICS			66.7%	0.45	0.67	0.53

The other performance matrices, precision, recall, F1-score, of both the datasets follow similar performance trends as the accuracy for comparison of algorithms.

### Summary

The results of the supervised learning classification algorithm are better than the previously reported results (26, 34) using the same datasets, see Table 6. As both datasets have real-time physiological signals, there are some outliers and noisy signals within the signal. Thus, intense pre-processing and outlier detection was performed to cleanse the dataset for better training of the classification algorithm. The achievement of better results than the previously published results reflects that our performed pre-processing step (thresholding and filtering) does help in developing a better classification model.

**Table 6:** Results comparison of supervised learning algorithms on datasets with previously reported work.

**Datasets**	**Classifier type**	**Ref**	**Feature**	**Highest reported classification accuracy**	**Highest achieved classification accuracy [this study] with 70-30% split**	**Highest achieved classification accuracy [this study] with K-fold validation**
Stress recognition in automobile drivers dataset		Table 5.8 of (23)	Respiratory rate	62.2%	66.8%	63.9%
	Supervised learning algorithms		Heart rate	52.6%	61.9%	59.9%
SWELL-KW dataset		Table 4 of (31)	Heart rate	64.1%	74.8%	75.0%

**Figure 2:**
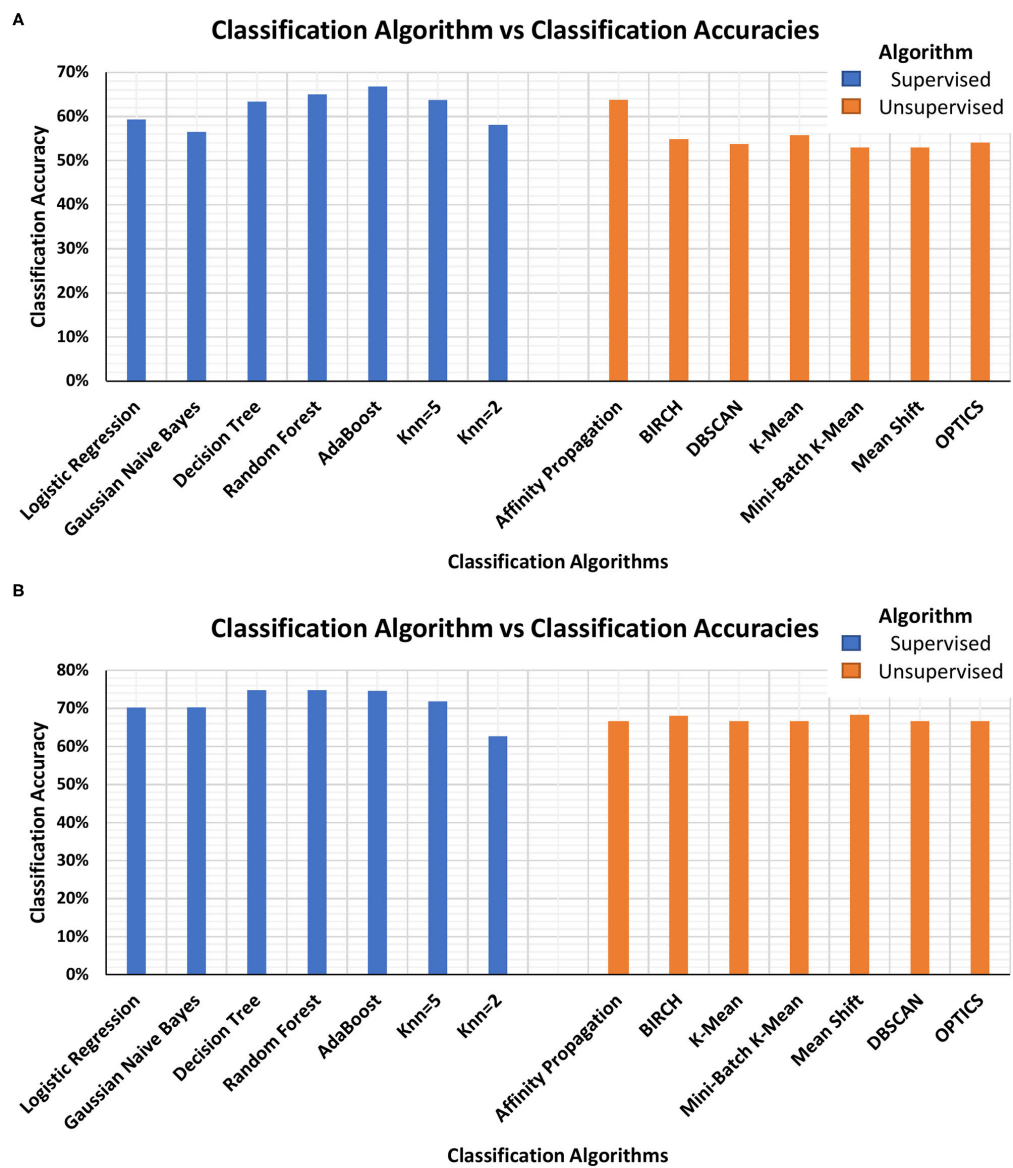
Bar-plot of classification accuracies of supervised and unsupervised classification algorithms using **(A)** Stress Recognition in Automobile Drivers Dataset and **(B)** SWELL-KW Dataset.

The authors acknowledge that these accuracies are not indicative of good performance but motivate the researchers to propose better supervised as well as unsupervised learning classification models for improved stress monitoring. Figure 2 shows the bar plot of classification accuracies of supervised and unsupervised classification algorithms using Stress Recognition in Automobile Drivers Dataset (Figure 2A) and SWELL-KW Dataset (Figure 2B). The use of an unsupervised classifier is important for the development of a non-invasive, robust, and continuous stress monitoring device since labeling the physiological signal in the ambulatory environment is a difficult and inaccurate task. The results in Tables 2-5 show the comparison of classification efficiencies of supervised and unsupervised classification algorithms. The difference in the highest classification accuracies is comparable, i.e., for Stress Recognition in Automobile Drivers dataset is 1% for respiratory rate-based model and 3% for two feature-based models. While for the SWELL-KW dataset, the difference is 6.5%. The overall accuracies of the supervised classifiers are better than the unsupervised classifier but as an unsupervised machine learning classifier does not require any intense pre-training as well as stress/non-stress labels, these results are encouraging the researchers to use the unsupervised models in stress monitoring wearable devices. Further improvements in unsupervised algorithms to optimize use in stress monitoring can potentially provide even better detection accuracies.

## Conclusion

Stress detection in a real-world environment is a complex task as labeling of the collected physiological signals is often inaccurate or non-existing. The questionnaires and self-reports are considered the only established way of getting the reference state of the participant emotion. The supervised machine learning classifiers have been able to accurately classify the stress state from the non-stress state. The problem of stress level labeling has already been reported in many studies but has rarely been addressed.

One possible solution is the use of an unsupervised machine learning classifier as such algorithms do not require labeled data. In this study, we have implemented different unsupervised classification algorithms to explore the feasibility of unsupervised stress detection and monitoring in different stress monitoring scenarios. For comparison, a set of different supervised learning algorithms was also implemented.

We have also performed an analysis to investigate the significant difference in the model performance using the standard deviations and confidence intervals. The performance of some models differs significantly from others. For instance, the performances of decision tree classifiers compared to k-nearest neighbors (k = 2) on Stress Recognition in Automobile Drivers dataset and random forest classifier compared to logistic regression classifier on SWELL-KW dataset are quite different. This leads us to the conclusion that a careful selection of classification models is required when aiming to develop an accurate stress detection system. The selection of the classifier is dependent on the type and shape of the data. It also depends upon the number of data points within the dataset.

The classification results indicate that unsupervised machine learning classifiers can show good performance in terms of classification accuracy, precision, recall and F1-score, without any training phase which is usually time-consuming and inaccurate. The findings enhance our understanding of the feasibility of unsupervised learning classifiers in wearable devices. Furthermore, these findings also may inform further approaches for the detection and monitoring of stress in an ambulatory environment.

## Data Availability Statement

The original contributions presented in the study are included in the article/supplementary material, further inquiries can be directed to the corresponding author/s.

## Author Contributions

TI, AS, and WW: conceptualization. TI and AS: methodology. TI, AE, and AS: validation. TI: formal analysis, investigation, writing—original draft preparation, and visualization. AE, AS, and WW: writing—review and editing. WW and AS: supervision. WW: project administration and funding acquisition. All authors read and agreed to the published version of the manuscript.

## Funding

The research leading to this publication was funded by the Science Foundation Ireland Research Professorship Award to WW (grant no. 15/RP/2765). AS acknowledges financial support from the University of Birmingham Dynamic Investment Fund.

## Conflict of Interest

The authors declare that the research was conducted in the absence of any commercial or financial relationships that could be construed as a potential conflict of interest.

## Publisher's Note

All claims expressed in this article are solely those of the authors and do not necessarily represent those of their affiliated organizations, or those of the publisher, the editors and the reviewers. Any product that may be evaluated in this article, or claim that may be made by its manufacturer, is not guaranteed or endorsed by the publisher.
